# Combined single- and dual-energy CT workflow for dose calculation in radiotherapy

**DOI:** 10.2340/1651-226X.2025.43827

**Published:** 2025-08-18

**Authors:** Hella Sand, Jens Edmund, Ane Appelt, Patrick Wohlfahrt, Vicki Trier Taasti, Laurids Østergaard Poulsen, Jimmi Søndergaard, Martin Skovmos Nielsen

**Affiliations:** a Department of Medical Physics, Aalborg University Hospital, Aalborg, Denmark; b Department of Clinical Medicine, Aalborg University, Aalborg, Denmark; c Department of Oncology, Radiotherapy Research Unit, Copenhagen University Hospital – Herlev and Gentofte, Herlev, Denmark; d Leeds Institute of Medical Research, University of Leeds, Leeds, UK; e Siemens Healthineers, Varian, Cancer Therapy Imaging, Forchheim, Germany; f Danish Centre for Particle Therapy, Aarhus University / Aarhus University Hospital, Aarhus, Denmark; g Department of Oncology, Aalborg University Hospital, Aalborg, Denmark

**Keywords:** radiotherapy planning, imaging, computed tomography, phantom, algorithm

## Abstract

**Background and purpose:**

Dual-energy computed tomography (DECT) is increasingly used in radiotherapy delineation due to its enhanced soft tissue contrast. DECT also supports direct dose calculation. However, as most current DECT scanners allow for use in only certain body regions, conventional single-energy computed tomography (SECT) is still needed for some patients. A safe clinical introduction of DECT thus requires a combined workflow. This study therefore investigates whether a unified Hounsfield look-up table (HLUT) can be applied across SECT and DECT reconstructions.

**Patient/material and methods:**

A Gammex Advanced Electron Density phantom containing tissue-equivalent inserts was scanned using SECT (70–140 kVp and Sn100–Sn140 kVp, Sn meaning tin-filtered) and dual-spiral DECT to identify matching HLUTs for three SECT methods, including a standard reconstruction (only 120 kVp; Method 1), and kVp-independent reconstructions providing mass density (MD; Method 2) or relative electron density (RED; Method 3). Dose agreement was subsequently tested on two anthropomorphic phantoms. For each SECT method, DECT reconstructions were compared through voxel-wise analysis of computed tomography (CT) numbers, and by performing dose calculations in three anatomical regions: head, thorax, and abdomen/pelvis.

**Results:**

Across all three SECT methods, DECT reconstructions with acceptable clinical CT number agreement were identified. Corresponding dose calculations between SECT- and DECT-based plans showed minimal differences.

**Interpretation:**

This phantom study demonstrates that a unified HLUT can be applied across SECT and DECT using standard 120 kVp, MD, or RED reconstructions. This approach may streamline clinical workflows and support a safe and practical transition to DECT-based treatment planning.

## Introduction

In radiotherapy (RT) treatment planning, computed tomography (CT) scans serve as the prime imaging modality with a dual purpose: delineation of tumors and organs-at-risk (OARs) and dose calculation [1]. The current standard is single-energy computed tomography (SECT), which operates at a single tube potential (typically 120 kVp) and provides high anatomical contrast between tissues with different densities, such as bone and airways [2]. However, SECT has limited ability to distinguish between different soft tissues like muscle, fat, and tumors.

One strategy to improve SECT image quality is adapting scan protocols to patient anatomy by selecting optimized tube potentials. Lower tube voltages (~70–80 kVp) improve contrast in smaller or less dense patients, while higher tube voltages (~130–140 kVp) reduce noise and artifacts in larger patients [3]. Accurate dose calculation, however, depends on precise conversion of CT numbers to relative electron density (RED) or mass density (MD) using Hounsfield look-up tables (HLUTs) [4]. Since CT numbers in Hounsfield units (HU) are energy-dependent, each tube voltage setting requires a specifically calibrated HLUT, increasing clinical complexity and the risk of applying incorrect HLUTs during dose calculation.

An attractive alternative to multiple voltage-dependent HLUTs is the Direct Density (DD) reconstruction algorithm (Siemens Healthineers, Forchheim, Germany), which generates quantitative images in RED (DDe) or MD (DDm) units [5–7]. These reconstructions are designed to be tube voltage-independent, allowing for optimal SECT image quality across patients without requiring multiple HLUTs.

To improve soft tissue contrast dual-energy computed tomography (DECT) has been proposed [8]. DECT is already well-established in diagnostic imaging [9], and is increasingly explored in RT as a potential alternative to MRI for tumor and OAR delineation [10–12]. Dual-spiral DECT operates at two distinct tube potentials, typically low-energy (~80 kVp) and high-energy (~140 kVp) [13], and enables advanced reconstructions such as virtual monoenergetic images (VMIs) and RED maps [14–16].

Integrating DECT into RT introduces mixed SECT and DECT workflows, as DECT may not be feasible in body regions affected by motion, time-resolved 4DCT or contrast-enhancement using bolus tracking [17]. In general, SECT and DECT require separate HLUTs due to differences in spectral and reconstruction characteristics, complicating clinical implementation of DECT by introducing additional HLUTs to establish and maintain, as well as increasing the risk of applying incorrect HLUTs during dose calculation.

Several studies have evaluated DECT reconstructions (VMIs, RED, Virtual Non-Contrast) for dose calculation across various tumor sites, comparing them to standard 120 kVp SECT-based plans [16, 18–21]. However, to our knowledge, no studies have explored combining SECT and DECT reconstructions to support a flexible clinical workflow with a unified HLUT for dose calculation.

This study aims to develop a robust, fail-safe method for applying a unified HLUT across both SECT- and DECT-based workflows, ensuring consistent dose calculations, optimizing treatment safety, improving image quality and streamlining RT planning.

## Material and methods

This is an experimental phantom study. No clinical patient data were used, and the EQUATOR guidelines were not applicable.

### Phantom setup and scanning protocol

**Figure 1:**
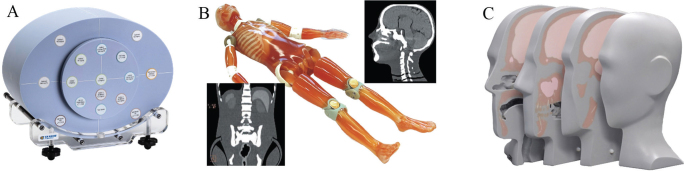
(A) Gammex Advanced Electron Density phantom (Sun Nuclear – A Mirion Medical Company, Middelton, WI, USA). (B) Anthropomorphic whole-body phantom (PBU-60, Kyoto Kagaku, Kyoto, Japan). (C) Head-and-neck phantom (731-HN, CIRS, Sun Nuclear – A Mirion Medical Company, Norfolk, VA, USA).

A Gammex Advanced Electron Density phantom (Sun Nuclear – A Mirion Medical Company, Middelton, WI, USA; Figure 1A) simulating a head and body geometry of an adult and containing nine tissue-equivalent inserts with respect to X-ray interactions (mass densities of 0.525–1.924 g/cm^3^, Supplementary Material, Table S1) was used in this study. To cover higher densities, an aluminum insert (MD = 2.71 g/cm^3^) was used. The Gammex phantom was scanned using a 16-bit CT scanner (SOMATOM go.Open Pro, Siemens Healthineers, Forchheim, Germany; version VB20) to generate HLUTs for both SECT and DECT acquisitions. A step-by-step guide (step 1–3) was followed [22], though without separate CT scans for each bone insert, and the HLUTs were generated by linear interpolation of the CT numbers and RED for the individual inserts.

### Single-energy CT acquisition, reconstruction methods and HLUT generation

SECT scans were acquired for both the head and body geometry. To cover variations in treatment planning practice in RT, three distinct SECT reconstruction methods were implemented for HLUT generation. Method 1: 120 kVp standard reconstruction (Siemens Qr40 kernel); Method 2: DDm (Siemens Sm40 kernel) reconstruction and Method 3: DDe (Siemens Sd40 kernel) reconstruction.

The energy spectra used for DD reconstructions ranged from 70 kVp to 140 kVp, and from Sn100 kVp to Sn140 kVp in 10 kVp increments for the body geometry. For the head geometry 80, 120 and Sn140 kVp were used. The ‘Sn’ designation refers to the use of a tin (Sn) filter placed in front of the X-ray tube, which removes low-energy photons from the beam, resulting in a harder energy spectrum compared to acquisitions without the Sn filter.

Beam hardening correction (BHC) [23] for bone was applied to all SECT and DECT reconstructions, minimizing differences between HLUTs derived from the head and body geometry. For Method 1, the SECT-based HLUT was generated as the average of CT numbers obtained from 120 kVp scans of the head and body geometry, respectively. For Method 2 and 3, the DDm- and DDe-based HLUTs were each constructed by averaging CT numbers from scans in both geometries across all the energy spectra in the range of 70 kVp to Sn140 kVp.

### Dual-energy CT acquisition, reconstruction methods and HLUT generation

DECT scans were acquired using 80 kVp/Sn140 kVp in head mode and 100 kVp/Sn140 kVp in body mode, DECT reconstructions included VMIs (at energies of 40–190 keV in steps of 10 keV overall, and 1 keV steps in selected energy ranges of interest), RED, and DDm/DDe images generated from the high-energy spectrum only (Sn140 kVp) for both head and body modes.

In this study, the term ‘CT number’ in HU is used as a general term to describe voxel values of a CT image reconstruction. RED, DDm, and DDe are linear scaled densities: RED/DDm/DDe = (‘voxel value’ / 1,000) + 1.

### Validating matching HLUTs on the Gammex phantom

For each of the three SECT methods, the corresponding optimal DECT-based HLUT was identified by calculating the root mean square error (RMSE) between the CT numbers for each of the nine tissue-equivalent inserts in the Gammex phantom in the SECT and DECT reconstructions:


$$RMSE\;=\sqrt{\frac{1}{n}\overset{n}{\underset{i=1}{\sum }}{\left(x_{i}-y_{i}\right)}^{2}}$$ (Eq. 1)

where *x* and *y* refer to the CT numbers measured in the inserts with SECT and DECT, respectively, *i* refers to the inserts, and *n* is the number of inserts (*n* = 9).

The DECT HLUT resulting in the lowest RMSE was considered the best match.

### Validating matching HLUTs on anthropomorphic phantoms

To validate the proposed approach in a clinically relevant setting, an anthropomorphic whole-body phantom (PBU-60, Kyoto Kagaku, Kyoto, Japan; Figure 1B) and a head-and-neck phantom (731-HN, CIRS, Sun Nuclear – A Mirion Medical Company, Norfolk, VA, USA; Figure 1C) were scanned in SECT and DECT mode using the same protocols as described in the previous sections with a CTDI_vol,16cm_ of ~55 mGy (head), CTDI_vol,32cm_ ~4 mGy (thorax), and CTDI_vol,32cm_ ~10 mGy (abdomen/pelvis) for both SECT and DECT. The scans were reconstructed with a slice thickness of 1 mm using Method 1, Method 2, and Method 3 for SECT, as well as the identified best-matching DECT reconstructions. The image resolution was 512 × 512 pixels and the reconstruction field-of-view was set to 600 × 600 mm^2^ for the whole-body phantom and 300 × 300 mm^2^ for the head-and-neck phantom.

**Figure 2:**
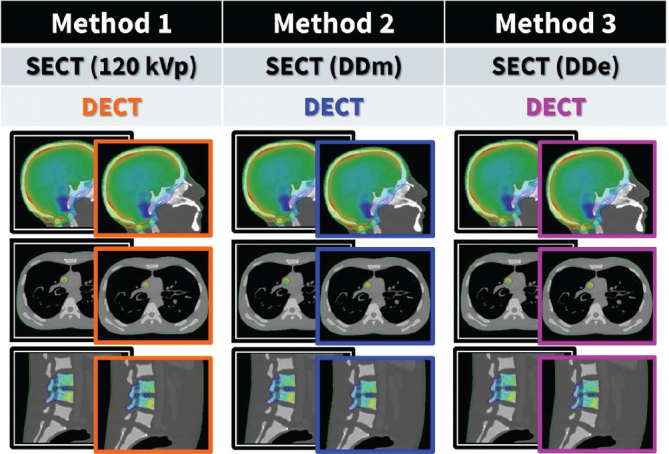
In three methods the correspondence of matched pairs of single-energy computed tomography (SECT) and dual-energy computed tomography (DECT) were tested in relation to dose calculation in three anatomical regions (head, thorax, abdomen/pelvis).

For the whole-body phantom, three clinical target volumes (CTVs) were delineated to simulate common tumor sites in the brain, lung, and lumbar spine (L3–L4). A single CTV was delineated in the brain of the head-and-neck phantom. The CTVs were delineated on the SECT image from Method 1 and rigidly copied to all six image sets, corresponding to the three matched pairs of SECT and DECT reconstructions (Figure 2).

For each CTV defined in the phantoms, a SECT-based treatment plan was created using each of the three SECT reconstruction methods (Method 1, Method 2, and Method 3), each with its associated HLUT. The treatment plans were performed using the Eclipse treatment planning system (version 18.0, Varian Medical Systems, Palo Alto, CA, USA), employing the Acuros XB algorithm, for three clinically relevant treatment scenarios: brain (palliative: 20 Gy in 4 fractions, with the CTV mean dose normalized to the prescribed dose; 3D conformal RT with two lateral beams), lung (curative: 66 Gy in 33 fractions, with the CTV mean dose normalized to the prescribed dose; IMRT with six beams), and L3–L4 of the lumbar spine (stereotactic body RT: 37.5 Gy in 3 fractions, with 95% of the prescribed dose covering the CTV and with no normalization; VMAT with three full arcs). These SECT-based plans were then re-calculated on the best-matching DECT reconstructions using the same HLUTs as in the original SECT treatment plans (Figure 2).

### CT number assessment in the anthropomorphic phantoms

Pairwise scatter plots were generated to visualize voxel-wise CT number correspondence between SECT and DECT reconstructions for all three methods and all evaluated anatomical sites. The CT numbers in the SECT scans were grouped into 50 HU bins, ranging from –1,000 HU to 2,000 HU, and then averaged within each bin. The CT numbers in the DECT scans in voxels corresponding to each bin in the SECT scan were similarly binned, and the averaged CT number from each bin in the SECT and DECT scan were plotted against each other. Bins containing fewer than 20 voxels (corresponding to < 0.02 cm^3^) were discarded and excluded from subsequent analysis. To evaluate agreement between the pairwise scatter plots, RMSEs were calculated using (Eq. 1) with *i* representing the bins and *x* and *y* taken as the averaged CT numbers in each bin.

### Dose distribution assessment in the anthropomorphic phantoms

Bland-Altman plots were generated to visualize voxel-wise dose agreement between SECT and DECT reconstructions across all methods and anatomical sites. The datapoints in the plots were generated by grouping the voxels in the SECT dose distribution into 0.5 Gy bins, and averaging the dose within each bin as well as averaging the dose from the corresponding voxels in the DECT dose distribution. To evaluate the agreement between the dose in the pairwise dose bins, RMSEs were calculated using Eq. (1) with *i* representing the bins and *x* and *y* taken as the averaged dose in each bin.

For each anatomical site, the binned SECT–DECT dose differences in percentages of prescribed doses were further grouped into boxplots for voxels receiving > 10% of the prescribed dose (Th10%) representing the low-dose volume and > 50% of the prescribed dose (Th50%) representing the high-dose volume.

For each CTV and method, 3D gamma analysis [24] was performed using the SECT-based dose distribution as the reference. The gamma criteria were set to 1 mm distance-to-agreement, 1% dose difference, using local normalization (i.e. 1% of the SECT-based dose at each voxel) and applying a dose threshold of 10% of the maximum dose in the SECT-based dose distribution.

High-density volumes (> 1.924 g/cm³) were < 1.0 cm³ in the whole-body phantom and < 4.5 cm³ in the head-and-neck phantom (mainly in the teeth). None of the high-density volumes were within the CTVs, and < 0.1 cm³ fell inside the Th10% volumes. Thus, the aluminum insert in the HLUTs had minimal impact on the dose calculations.

## Results

### Best matching HLUTs

**Figure 3:**
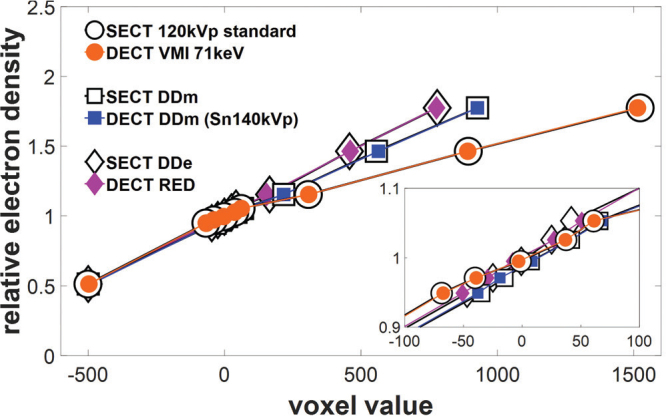
Best-matching HLUTs for all three methods plotted as RED versus average voxel values, that is CT numbers in Hounsfield units (HU) for Method 1, scaled mass densities (Method 2), and scaled relative electron densities (Method 3). The datapoints correspond to the Gammex phantom inserts. Method 1: circles; Method 2: squares; Method 3: diamonds. *Inset (lower right corner):* Zoomed view of the soft tissue region around 0 HU. SECT: single-energy computed tomography; DECT: dual-energy computed tomography; HLUT: Hounsfield look-up table; VMI: virtual monoenergetic images; RED: relative electron density.

**Figure 4:**
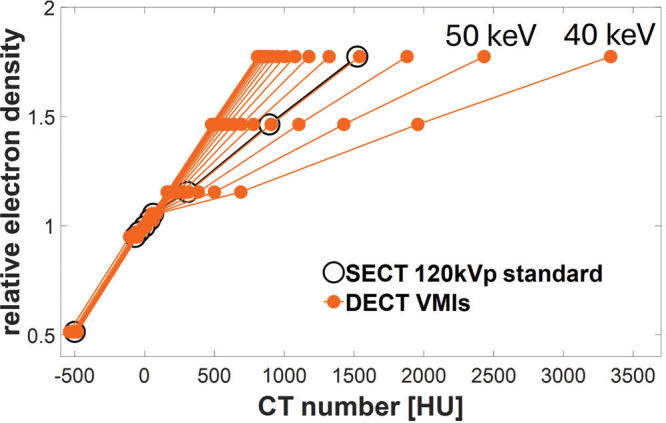
DECT-based HLUTs from VMI reconstructions, at energies of 40–190 keV in steps of 10 keV and the HLUT for the SECT 120 kVp standard reconstruction (Method 1). CT: computed tomography; SECT: single-energy computed tomography; DECT: dual-energy computed tomography; HLUT: Hounsfield look-up table; HU: Hounsfield units; VMI: virtual monoenergetic images.

The best-matching HLUTs for all three methods are illustrated in Figure 3 with the RED of the Gammex phantom inserts plotted against the average CT numbers. For the SECT standard reconstruction (Method 1), the corresponding optimal DECT-based HLUT was identified among the VMI reconstructions (Figure 4). The VMI at 71 keV demonstrated the best agreement, yielding the lowest RMSE over the nine tissue-equivalent inserts of 4 HU. The DECT-derived DDm from Sn140 kVp (Method 2) most closely matched the SECT DDm, with an RMSE of 5 HU. The DECT RED provided the closest correspondence for the SECT DDe (Method 3), with an RMSE of 6 HU. An overview of the RMSE calculations for all three methods across all evaluated DECT reconstructions is provided in Supplementary Material, Table S2.

### CT number and dose evaluations in the anthropomorphic phantoms

**Figure 5:**
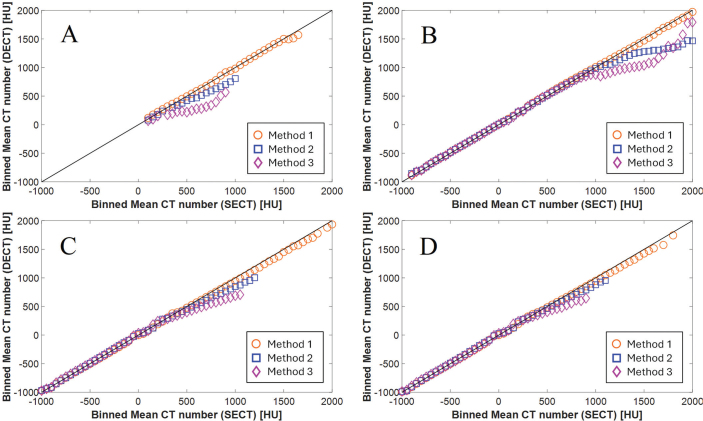
Pairwise scatter plots visualizing voxel-wise CT number correspondence in 50 HU bins between SECT and DECT reconstructions. (A) Head of whole-body phantom. (B) Head-and-neck phantom. (C) Thorax of whole-body phantom. (D) Abdomen/pelvis of whole-body phantom. Black lines: Identity lines. CT: computed tomography; SECT: single-energy computed tomography; DECT: dual-energy computed tomography; HU: Hounsfield units.

Figure 5 shows voxel-wise scatter plots comparing CT numbers between SECT and the best matching DECT for the whole-body phantom and the head-and-neck phantom across all three methods. In the head region for Method 1, the scatter points align closely with the identity line, indicating good agreement, with RMSEs of 27 HU for the whole-body phantom and 21 HU for the head-and-neck phantom. For Method 2, deviations are seen for CT numbers above ~500 HU (whole-body phantom) and ~1,100 HU (head-and-neck phantom), where SECT values exceed DECT with RMSEs of 127 HU and 183 HU, respectively. Method 3 shows larger discrepancies, with deviations above ~300 HU (whole-body phantom) and ~900 HU (head-and-neck phantom), with RMSEs of 279 HU and 216 HU, respectively.

Trends in the scatter plots for the thorax and abdomen/pelvis region mirror those from the head region: Method 1 shows the best agreement, Methods 2 and 3 moderate and larger deviations. RMSEs for thorax were 42 HU, 53 HU, and 90 HU, and for abdomen/pelvis 43 HU, 78 HU, and 124 HU for Methods 1, 2, and 3, respectively.

**Figure 6:**
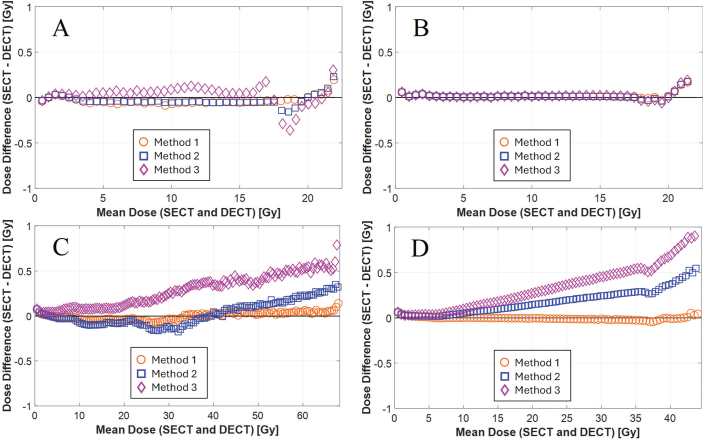
Bland–Altman plots visualizing voxel-wise dose correspondence in 0.5 Gy bins between dose calculations performed on SECT and DECT reconstructions. The x-axis (Mean Dose) represents the mean of the SECT and DECT dose values for the voxels in each bin. (A) Head of whole-body phantom. (B) Head-and-neck phantom. (C) Thorax of whole-body phantom. (D) Abdomen/pelvis of whole-body phantom. SECT: single-energy computed tomography; DECT: dual-energy computed tomography.

Figure 6 presents Bland–Altman plots of voxel-wise dose differences between SECT and the best matching DECT. For the whole-body phantom in the head region, Methods 1 and 2 show small negative offsets (< 0.1 Gy), indicating slightly higher DECT dose estimates. At ~18–22 Gy, Method 2 shifts towards positive deviations up to ~0.2 Gy. Method 3 shows a consistent positive offset (~0.1 Gy) with deviations increasing to ± 0.4 Gy around 18–22 Gy. In contrast, for the head-and-neck phantom, all methods align closely with the zero line, indicating good agreement, with only minor upward trends (< 0.2 Gy) around 18–22 Gy. The RMSEs for the whole-body phantom were 0.06 Gy for Method 1, 0.07 Gy for Method 2, and 0.11 Gy for Method 3. For the head-and-neck phantom, the RMSEs were 0.04 Gy for Method 1 and 2, and 0.05 Gy for Method 3.

Trends in the Bland–Altman plots for the thorax and abdomen/pelvis in the whole-body phantom mirror those from the head region: Method 1 remains close to zero, while Methods 2 and 3 show increasing positive deviations. In the thorax, Method 2 stays within 0.4 Gy, whereas Method 3 exceeds 0.5 Gy. In the abdomen/pelvis, deviations reach ~0.5 Gy for Method 2 and up to nearly 1 Gy for Method 3, indicating increasing SECT-DECT discrepancy. RMSEs for the thorax were 0.04 Gy for Method 1, 0.14 Gy for Method 2, and 0.34 Gy for Method 3; and for the abdomen/pelvis 0.02 Gy for Method 1, 0.22 Gy for Method 2, and 0.41 Gy for Method 3.

**Figure 7:**
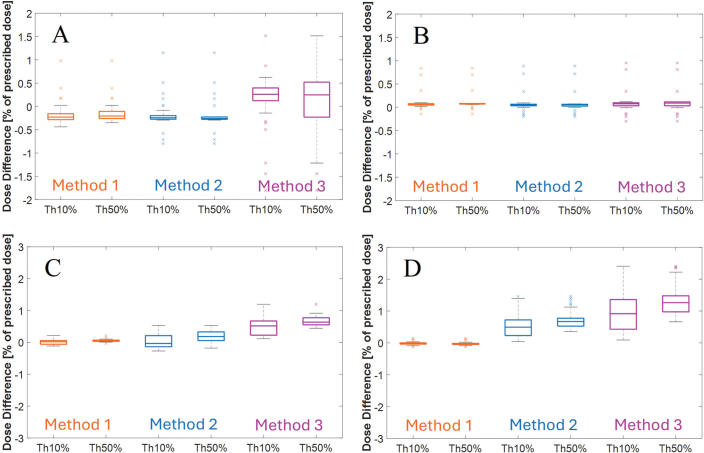
Boxplots visualizing dose differences between SECT and DECT for the dose volume receiving > 10% of the prescribed dose (Th10%), and the dose volume receiving > 50% of the prescribed dose (Th50%). (A) Head of whole-body phantom. (B) Head-and-neck phantom. (C) Thorax of whole-body phantom. (D) Abdomen/pelvis of whole-body phantom. In the boxplots, the central line indicates the median, the box edges represent interquartile range (IQR), and the whiskers extend to 1.5 times the IQR. Data points outside this range are plotted individually as outliers. SECT: single-energy computed tomography; DECT: dual-energy computed tomography.

Figure 7 shows boxplots of dose differences between SECT and the best matching DECT within Th10% and Th50% volumes. For the whole-body phantom in the head region, Methods 1 and 2 show median differences around –0.2% and all outliers below ± 1.2%. Method 3 shows a positive median difference (~0.3%) and outliers up to ± 1.8%. Similar trends are seen in the boxplots for the whole-body phantom in the thorax and abdomen/pelvis regions, though Method 3 shows more pronounced outliers, with dose differences exceeding 2.0% in the abdomen/pelvis region. For the head-and-neck phantom, all methods show negligible differences: medians near 0% and only a few positive outliers up to 1.0%, confirming high consistency between SECT and DECT in this phantom.

Gamma analysis performed within the CTVs yielded 100% passing rates across all three methods and anatomical sites for both phantoms, using the evaluation criteria of 1 mm distance-to-agreement, 1% dose difference (local normalization), and a 10% dose threshold. This confirms high spatial and dose agreement between SECT- and DECT-based dose distributions within target regions.

## Discussion and conclusion

Combined SECT and DECT workflows are becoming more frequently encountered in RT and are likely to further increase in the future. This study aimed to develop a robust, fail-safe method for applying a unified HLUT applicable to both SECT- and DECT-based workflows. The approach was designed to ensure consistent dose calculation across SECT and DECT reconstructions, thereby optimizing treatment safety, improving image quality, and streamlining RT workflows. By eliminating the need for separate HLUTs for SECT and DECT, the method can reduce the workload associated with establishing and maintaining multiple HLUTs and minimize the risk of incorrect HLUT assignment during dose calculation.

Method 1 (standard 120 kVp, SECT) paired with a 71 keV VMI showed the best SECT–DECT agreement, with minimal RMSE values and negligible dose differences. These results align with Ohira et al. [18], who found a VMI at 77 keV had the lowest CT number differences relative to 120 kVp images, when using a single-source DECT scanner with rapid kilovolt peak switching from GE Medical Systems. It should be noted that HLUTs for standard reconstructions vary as a function of tube potential [6]. However in this study, Method 1 was limited to SECT acquired at a standard tube potential of 120 kVp. Other tube potentials are increasingly used in clinical SECT workflows due to the possibility of automatic tube potential selection in modern CT scanners [25]. For each SECT tube potential, a corresponding optimal DECT reconstruction must be identified, if using standard reconstructions.

Method 2 (DDm, SECT) combined with a DECT-based DDm (Sn140 kVp) also showed acceptable agreement. Minor deviations in denser regions did not result in clinically relevant dose differences, supporting its compatibility with a single HLUT. For Method 3 (DDe, SECT), although showing a low RMSE value of 6 HU in the HLUT comparison also showed dose deviations exceeding 2% of the prescribed dose in the pelvic region on the whole-body phantom, at the limit of the clinical acceptability threshold [26]. This likely reflects material discrepancies, as the phantom’s epoxy resin-based bones have densities (1.31 g/cm^3^ and 1.11 g/cm^3^) notably lower than real bone (up to 1.92 g/cm^3^) [27]. Also, the elemental composition of epoxy resin is not tissue-equivalent. Such mismatches may affect the accuracy of both DDe and RED imaging in different ways and increase discrepancies, due to their shared reliance on the assumption of tissue equivalence.

However, all methods produced clinically acceptable agreement for the head-and-neck phantom, which better mimics human tissues, including cortical bone at 1.91 g/cm^3^. The CT number scatter plot in Figure 5B shows deviation for CT numbers above ~800 HU (teeth), but this region was outside the Th10% volume, thus not affecting the Bland–Altman plot for the dose differences (Figure 6B). Deviations above 18 Gy are likely due to edge effects near the skull’s periphery.

For Method 3, the unrealistic material properties of the whole-body phantom is considered to be the dominant factor in the observed discrepancies between the SECT-based DDe and the DECT-based RED methods. This interpretation is supported by the good agreement obtained for the head-and-neck phantom, which more closely resembles human tissue composition, and by the low RMSE in the HLUT comparison performed on tissue-equivalent density inserts with respect to X-ray interactions in the Gammex phantom. These findings emphasize limitations of non-tissue-equivalent phantoms for validating quantitative imaging like DDe and RED, and suggest that the results for Method 3 in the whole-body phantom are unlikely to reflect the true clinical performance. This is consistent with the work of Zhu et al. [16], who showed that RED-based dose calculations from spectral CT were comparable to conventional CT across various regions.

Overall, this study supports the feasibility of integrating DECT into existing RT workflows using SECT-based HLUTs, without compromising the consistency of dose calculation relative to current clinical practice, provided that HLUTs are carefully selected and validated for the specific SECT acquisition settings (tube potential, filtration etc.) and reconstruction method used. The identified best-matching HLUTs in this study reflect realistic clinical imaging settings, making the approach readily implementable in practice. While Methods 2 and 3 (DDe and DDm) are specific to Siemens DECT implementations, Method 1 (VMI-based SECT approach) will be applicable across different DECT vendors and technologies, the optimal VMI energy may differ from the 71 keV identified in this study.

As a next step, this methodology should be tested on real patient data to assess its robustness in clinical variability, including differences in patient anatomy, positioning, and tissue composition. Patient-based validation will help determine whether the proposed unified HLUT strategy can maintain dose accuracy across diverse clinical scenarios. Future investigations should also consider the influence of contrast agents, metal implants, and image artifacts on HLUT matching and resulting dose distributions.

## Supplementary Material



## Data Availability

Data are available on reasonable request. For more information contact the corresponding author.

## References

[1] Gardner SJ, Kim J, Chetty IJ. Modern radiation therapy planning and delivery. Hematol Oncol Clin North Am. 2019;33:947–62. 10.1016/j.hoc.2019.08.005 31668213

[2] Goldman LW. Principles of CT and CT technology. J Nucl Med Technol. 2007;35:115–30. 10.2967/jnmt.107.042978 17823453

[3] Kang P, Liao M, Wester MR, Leeder JS, Pearce RE. Standardization and optimization of computed tomography protocols to achieve low-dose. Ratio. 2014;36:490–9. 10.1016/j.jacr.2013.10.016

[4] Saini A, Pandey V, Kumar P, Singh A, Pasricha R. Investigation of tube voltage dependence on CT number and its effect on dose calculation algorithms using thorax phantom in Monaco treatment planning system for external beam radiation therapy. J Med Phys. 2021;46:315–23. 10.4103/jmp.JMP_124_20 35261502 PMC8853450

[5] Flatten V, Friedrich A, Engenhart-Cabillic R, Zink K. A phantom based evaluation of the dose prediction and effects in treatment plans, when calculating on a direct density CT reconstruction. J Appl Clin Med Phys. 2020;21:52–61. 10.1002/acm2.12824 32176455 PMC7075385

[6] van der Heyden B, Öllers M, Ritter A, Verhaegen F, van Elmpt W. Clinical evaluation of a novel CT image reconstruction algorithm for direct dose calculations. Phys Imaging Radiat Oncol. 2017;2:11–16. 10.1016/j.phro.2017.03.001

[7] Nelson G, Pigrish V, Sarkar V, Su FC, Salter B. Technical note: the use of DirectDensity TM and dual-energy CT in the radiation oncology clinic. J Appl Clin Med Phys. 2019;20:125–31. 10.1002/acm2.12546 PMC6414137 30851087

[8] Kruis MF. Improving radiation physics, tumor visualisation, and treatment quantification in radiotherapy with spectral or dual-energy CT. J Appl Clin Med Phys. 2022;23:1–17. 10.1002/acm2.13468 PMC8803285 34743405

[9] Tatsugami F, Higaki T, Nakamura Y, Honda Y, Awai K. Dual-energy CT: minimal essentials for radiologists. Jpn J Radiol. 2022;40:547–59. 10.1007/s11604-021-01233-2 34981319 PMC9162973

[10] Kraft J, Lutyj P, Grabenbauer F, Ströhle SP, Tamihardja J, Razinskas G, et al. Assessment of dual-energy computed tomography derived virtual monochromatic imaging for target volume delineation of brain metastases. Radiother Oncol. 2023;187:109840. 10.1016/j.radonc.2023.109840 37536377

[11] Noid G, Zhu J, Tai A, Mistry N, Schott D, Prah D, et al. Improving structure delineation for radiation therapy planning using dual-energy CT. Front Oncol. 2020;10:1–11. 10.3389/fonc.2020.01694 32984048 PMC7484725

[12] Taasti VT, Wohlfahrt P. From computed tomography innovation to routine clinical application in radiation oncology – a joint initiative of close collaboration. Phys Imaging Radiat Oncol. 2024;29:100550. 10.1016/j.phro.2024.100550 38390587 PMC10881422

[13] Johnson TRC. Dual-energy CT: general principles. AJR Am J Roentgenol. 2012;199:S3–8. 10.2214/AJR.12.9116 23097165

[14] McCollough CH, Leng S, Yu L, Fletcher JG. Dual- and multi-energy CT: principles, technical approaches, and clinical applications. Radiology. 2015;276:637–53. 10.1148/radiol.2015142631 26302388 PMC4557396

[15] Albrecht MH, Vogl TJ, Martin SS, Nance JW, Duguay TM, Wichmann JL, et al. Review of clinical applications for virtual monoenergetic dual-energy CT. Radiology. 2019;293:260–71. 10.1148/radiol.2019182297 31502938

[16] Zhu Q, Wei S, Wang Z, Xu H, Zhou B, Qu H, et al. Feasibility of dose calculation for treatment plans using electron density maps from a novel dual-layer detector spectral CT simulator. Radiat Oncol. 2024;19:1–9. 10.1186/s13014-024-02479-6 39049106 PMC11267670

[17] Yang M, Wohlfahrt P, Shen C, Bouchard H. Dual- and multi-energy CT for particle stopping-power estimation: current state, challenges and potential. Phys Med Biol. 2023;68:04TR01. 10.1088/1361-6560/acabfa 36595276

[18] Ohira S, Yagi M, Iramina H, Karino T, Washio H, Ueda Y, et al. Treatment planning based on water density image generated using dual-energy computed tomography for pancreatic cancer with contrast-enhancing agent: phantom and clinical study. Med Phys. 2018;45:5208–17. 10.1002/mp.13180 30198189

[19] Ohira S, Komiyama R, Karino T, Washio H, Ueda Y, Miyazaki M, et al. Volumetric modulated arc therapy planning based on virtual monochromatic images: effect of inaccurate CT numbers on dose distributions. Phys Medica. 2019;60:83–90. 10.1016/j.ejmp.2019.03.022 31000091

[20] Edmund J, Feen Rønjom M, van Overeem Felter M, Maare C, Margrete Juul Dam A, Tsaggari E, et al. Split-filter dual energy computed tomography radiotherapy: from calibration to image guidance. Phys Imaging Radiat Oncol. 2023;28:100495. 10.1016/j.phro.2023.100495 37876826 PMC10590838

[21] Noid G, Schott D, Paulson E, Zhu J, Shah J, Li XA. Technical note: using virtual noncontrast images from dual-energy CT to eliminate the need of precontrast CT for x-ray radiation treatment planning of abdominal tumors. Med Phys. 2021;48:1365–71. 10.1002/mp.14702 33386614

[22] Peters N, Trier Taasti V, Ackermann B, Bolsi A, Vallhagen Dahlgren C, Ellerbrock M, et al. Consensus guide on CT-based prediction of stopping-power ratio using a Hounsfield look-up table for proton therapy. Radiother Oncol. 2023;184:109675. 10.1016/j.radonc.2023.109675 37084884 PMC10351362

[23] Lifton JJ, Malcolm AA. Estimating the product of the x-ray spectrum and quantum detection efficiency of act system and its application to beam hardening correction. Sensors. 2021;21:3284. 10.3390/s21093284 34068586 PMC8126163

[24] Low DA, Harms WB, Mutic S, Purdy JA. A technique for the quantitative evaluation of dose distributions. Med Phys. 1998;25:656–61. 10.1118/1.598248 9608475

[25] Browne JE, Bruesewitz MR, Thomas V, Thomas KB, Hull NC, McCollough CH, et al. Procedure for optimal implementation of automatic tube potential selection in pediatric CT to reduce radiation dose and improve workflow. J Appl Clin Med Phys. 2021;22:194–202. 10.1002/acm2.13098 PMC7882104 33338314

[26] International Atomic Energy Agency. Absorbed Dose Determination in External Beam Radiotherapy, Technical Reports Series No. 398 (Rev. 1), IAEA, Vienna, 2024.

[27] International Commission on Radiation Units and Measurements. ICRU Report 44: Tissue substitutes in radiation dosimetry and measurement. Bethesda (MD): ICRU; 1989.

