# Supplementary material for: Combined single- and dual-energy CT workflow for dose calculation in radiotherapy
Source: Acta Oncol. 2025 Aug 18;64:43827. doi: 10.2340/1651-226X.2025.43827 (PMC12371750; doi:10.2340/1651-226X.2025.43827)
Supplement: Supplementary file 1 [file AO-64-43827-s1.pdf]

Supplementary material has been published as submitted. It has not been copyedited, or typeset by Acta Oncologica

Supplementary Material

Table S1: Data on the nine tissue-equivalent density inserts used in the study. *: Custom-made insert from PTW. **: Inserts from the Gammex Advanced Electron Density phantom.

Description	Physical Density (g/cm^3)	Electron Density Relative to Water
*Lung	0.525	0.514
**HE General Adipose	0.960	0.949
**HE Breast 50:50	0.985	0.971
**HE CT Solid Water	1.019	0.995
**HE Brain	1.052	1.026
**HE Liver	1.080	1.053
**HE Inner Bone	1.203	1.153
**$CaCO_3$ 50%	1.560	1.463
**HE Cortical Bone	1.924	1.774

Table S2: Overview of root mean square error (RMSE) calculations between the CT numbers of the nine Gammex phantom inserts in the SECT reconstruction and all DECT reconstructions assessed in this study, for each of the three methods. For all three SECT methods the three best matching DECT reconstructions are highlighted in bold. *Best matching HLUT. **Second best matching HLUT. ***Third best matching HLUT.

DECT reconstruction	Method 1<br>SECT 120 kVp<br>(HU)	Method 2<br>SECT DDm<br>(HU)	Method 3<br>SECT DDe<br>(HU)
VMI 40 keV	713	944	1005
VMI 50 keV	357	588	649
VMI 60 keV	141	371	432
VMI 70 keV	**8****	238	299
VMI 71 keV	**4***	228	288
VMI 72 keV	**13*****	218	278
VMI 80 keV	78	153	213
VMI 90 keV	135	96	156
VMI 100 keV	175	58	117
VMI 110 keV	203	32	89
VMI 120 keV	223	**18**/*****	69
VMI 130 keV	238	**18**/*****	54
VMI 140 keV	250	25	42
VMI 150 keV	259	32	34
VMI 160 keV	266	39	27
VMI 170 keV	272	44	22
VMI 180 keV	276	49	18
VMI 190 keV	280	52	**14*****
Sn140 kVp (DDm)	230	**5***	64
Sn140 kVp (DDe)	290	63	**7****
RED	292	64	**6***
